# Enhancing the color and stress tolerance of cherry shrimp (*Neocaridina davidi* var. *red*) using astaxanthin and *Bidens Pilosa*

**DOI:** 10.1371/journal.pone.0315585

**Published:** 2024-12-19

**Authors:** Wei-Wei Hou, Yu-Tzi Chang, Wen-Chin Yang, Hong-Yi Gong, Yen-Ju Pan, Te-Hua Hsu, Chang-Wen Huang

**Affiliations:** 1 Department of Aquaculture, National Taiwan Ocean University, Keelung, Taiwan; 2 Agricultural Biotechnology Research Center, Academia Sinica, Nankang, Taipei, Taiwan; 3 Center of Excellence for the Oceans, National Taiwan Ocean University, Keelung, Taiwan; Benha University, EGYPT

## Abstract

This study aimed to evaluate the effects of different concentrations of astaxanthin and *Bidens Pilosa* compound feed additives on the color and hypoxia tolerance of cherry shrimp (*Neocaridina davidi* var. red). Color parameters were assessed using CIELAB color space, and differential gene expression related to color and stress was analyzed using reverse transcription quantitative polymerase chain reaction (RT-qPCR) to understand the gene regulatory mechanisms affecting color expression and stability. Over a 56-day rearing period, the feed additives AX100 (astaxanthin 100 mg/kg) and AX100+BP (astaxanthin 100 mg/kg + *B*. *pilosa* 5 g/kg) significantly reduced the color difference values compared to the standard sample (ΔE*_ab_), indicating notable color boosting effects. This included a reduction in lightness (L*), a decrease in color hue angle (h*) with AX100, and an increase in redness (a*) and chroma (C*) with AX100+BP. We further designed 22 color-related gene primers, 16 of which amplified the target fragment. Six gene sets exhibited significant differences among all feed treatment groups and were correlated with color expression. After 9 hours of hypoxic stress, body color remained stable in the feed additive groups, especially in the AX100 + BP and AX200 + BP (astaxanthin 200 mg/kg + *B*. *pilosa* 5 g/kg) groups, with color differences before and after hypoxic stress remaining below the discernible threshold of the human eye, indicating optimal color stability. Additionally, the *CAT* gene, among the stress-related genes that successfully amplified, showed significant differences among feed treatment groups and correlated with color stability based on color difference values. In conclusion, the composite addition of 100 mg/kg astaxanthin and 5 g/kg *Bidens pilosa* (AX100 + BP) was identified as the most effective treatment. This formulation significantly enhanced cherry shrimp color, evidenced by improved parameters such as decreased lightness and increased redness. Moreover, AX100 + BP demonstrated superior color stability under hypoxic conditions, with ΔE*_ab_ values remaining below the discernible threshold of the human eye, highlighting its potential for maintaining optimal color during transportation. Offering a basis for enhancing the commercial value and reducing the sale risks of cherry shrimp.

## 1. Introduction

In recent years, ornamental shrimp has become popular in the international ornamental aquarium market [1], with Taiwan emerging as the primary supplier of these invertebrates. Approximately one in six ornamental shrimps worldwide are from Taiwan, with over 18 million annual exports since 2010 [2, 3]. Particularly the multi-colored *Neocaridina* shrimp, has garnered attention owing to its diverse color strains. Over decades of selective breeding, dozens of distinctly colored strains, including *Neocaridina davidi* var. *red* that is among the earliest modified and extensively researched strains, have been developed [4, 5]. The price of *N*. *davidi* var. *red* varies considerably based on the intensity of the red color on the body, making body color a pivotal determinant of its market value [6]. Many factors, including individual variation, environmental conditions, and dietary nutrients, influence the body color in shrimp [7].

Aquatic animals face various stressors related to transportation, fishing, confinement, density, air exposure, and changes in water quality [8]. Oxygen and water quality control during transportation are crucial for aquatic animals. Accumulation of carbon dioxide must be managed as these species are susceptible to hypoxic stress [9, 10]. Transportation stress affects various indicators, including muscle color in tilapia (Nile tilapia) [11] and *Oncorhynchus mykiss* [12], muscle texture, and pH. Stress tolerance can be improved using feed additives. For example, addition of carotenoids to the feed of carp (*Cyprinus carpio*) increases its resistance to ammonia-induced stress [13]. Moreover, addition of blunt tops *Arthrospira platensis* to the feed of yellow catfish (*Pelteobagrus fulvidraco*) reduces the hypoxic stress caused by air exposure [14]. Many herbaceous plants also reduce the effects of transportation, crowding, hypoxia, and temperature [15].

Crustaceans, including ornamental shrimps, cannot synthesize carotenoids endogenously and must obtain these pigments via their diet and metabolic transformation [16]. Carotenoids, particularly astaxanthin (AX), are crucial for enhancing the vibrant coloration in shrimp species. Due to its superior free radical scavenging capabilities [17, 18], astaxanthin is essential for improving pigmentation and overall health in ornamental aquariums [19, 20]. In aquaculture, the dietary inclusion of astaxanthin has been widely adopted due to its effectiveness in enhancing the red coloration of shrimp, thereby increasing their market value [21].

Owing to the increasing concerns about the safety of aquatic products, the use of many medicinal chemicals has been banned, leading to the utilization of Chinese herbal additives to protect against harmful stimuli [22]. Previous studies have confirmed the beneficial effects of Chinese herbal medicines as feed additives [23]. Specifically, *Bidens pilosa* (BP), a member of the *Asteraceae* family, has attracted global attention for its diverse pharmacological properties. To date, over 300 bioactive compounds have been identified in *B*. *pilosa* [24]. These compounds exert various effects, including anti-cancer, anti-inflammatory, anti-diabetic, antioxidant, immunomodulatory, anti-malarial, antibacterial, antifungal, blood pressure-lowering, vasodilation, and wound-healing [25–27]. *B*. *pilosa* has been studied as a feed additive for tilapia in aquaculture [28], *B*. *pilosa* can enhance the immune response, improve stress tolerance, and promote overall health in aquatic animals [29, 30]. Studies have shown that incorporating BP into shrimp diets not only aids in stress management during transportation and environmental changes but also enhances the pigmentation by influencing metabolic pathways associated with carotenoid utilization [31].

Currently, there are no reports on the integration of astaxanthin and *Bidens pilosa* in the diets of ornamental shrimp. This study explores their synergistic effects in enhancing color, stress resistance, and overall health. The research evaluates various concentrations of astaxanthin and *Bidens pilosa* on the body color and hypoxia tolerance of cherry shrimp (*N*. *davidi* var. *red*). Astaxanthin is known for its antioxidant properties and ability to improve pigmentation, while Bidens pilosa provides anti-stress and immune-modulating effects. This study combines colorimetric and molecular biology techniques to identify optimal feed formulations, improving the aesthetic appeal and marketability of ornamental shrimp.

## 2. Materials and methods

### 2.1 Ethical considerations

This study involved the use of Cherry shrimp (*Neocaridina davidi* var. *red*), which are classified as invertebrates and are therefore exempt from the requirement of Institutional Animal Care and Use Committee (IACUC) approval. Despite the absence of specific regulations mandating ethical approval for invertebrate research, all experimental protocols strictly adhered to rigorous standards designed to ensure the welfare of the specimens. These standards encompassed all aspects of feeding and stress experimenting procedures, thereby guaranteeing the proper management and humane treatment of the aquatic ornamental shrimp throughout the duration of the study.

### 2.2 Experimental species

For this study, Cherry shrimp (*N*. *davidi* var. *red*) was provided by private ornamental shrimp operators and consisted of juvenile all-male (♂) individuals (S1 Fig). The breeding temperature was maintained at approximately 24 °C. The breeding water was fully aerated tap water with total dissolved solids (TDS) of approximately 150–200 mg/L, and the daily light duration was 8 h. Pneumatic filtering equipment and vertical floating climbers, including mesh bags, were placed in the tank. The bottom layer was covered with approximately 1 cm of black soil. During the first 3 weeks of the experiment, all experimental groups were fed a standard diet (control diet) for acclimation.

### 2.3 Experimental feed

The feed for this experiment was homemade as previously described [32]. Feed additives included astaxanthin (Carophyll Pink 10% CWS; DSM Nutritional Product Ltd, Switzerland) and *B*. *pilosa* (Altruism Co., Ltd., Taiwan). Five experimental diets were used. The control group had no additional substances added to the feed. The remaining four groups contained the specific additives. The two treatment groups were supplemented with different concentrations of astaxanthin and labeled as AX100 (astaxanthin 100 mg/kg) and AX200 (astaxanthin 200 mg/kg). The final two treatment groups were treated with both astaxanthin and *B*. *pilosa* and labeled as AX100 + BP (astaxanthin 100 mg/kg + *B*. *pilosa* 5 g/kg) and AX200 + BP (astaxanthin 200 mg/kg + *B*. *pilosa* 5 g/kg; Table 1).

**Table 1:** Composition of experimental diets fed to cherry shrimp (*N*. *davidi* var. red) including varying concentrations of astaxanthin and *Bidens pilosa* extract over a 8-week period (g/100 g, *n* = 3).

Diet component^a^	Control	AX100	AX100 + BP	AX200	AX200 + BP
Wheat flour	31.6	31.5	31.0	31.4	30.9
Brown fish meal	23.6	23.6	23.6	23.6	23.6
Dehulled soybean meal	15.0	15.0	15.0	15.0	15.0
Whole soybean meal	5.0	5.0	5.0	5.0	5.0
Wheat gluten	3.0	3.0	3.0	3.0	3.0
Fish oil	2.0	2.0	2.0	2.0	2.0
Fermented soybean meal	5.0	5.0	5.0	5.0	5.0
CMC^b^	3.0	3.0	3.0	3.0	3.0
Cholesterol	0.5	0.5	0.5	0.5	0.5
Sodium glutamate	2.2	2.2	2.2	2.2	2.2
Disodium inosinate	0.3	0.3	0.3	0.3	0.3
Squid meal	3.0	3.0	3.0	3.0	3.0
Lecithin	1.0	1.0	1.0	1.0	1.0
Choline	0.5	0.5	0.5	0.5	0.5
Mineral mixture	4.0	4.0	4.0	4.0	4.0
Vitamin mixture	0.3	0.3	0.3	0.3	0.3
Astaxanthin (10%)	-	0.1	0.1	0.2	0.2
*Bidens pilosa*	-	-	0.5	-	0.5
Proximate composition	100	100	100	100	100

^a^ All components are measured on a dry weight basis.

^b^ CMC: Carboxymethyl cellulose.

### 2.4 Experimental conditions

#### 2.4.1 Feeding experiment

This experiment was performed with five feed-additive groups: Control, AX100, AX100 + BP, AX200, and AX200 + BP groups. For body color data, 20 shrimp were used per tank; for genetic data, 30 shrimp were used per tank, with three replicate sets for the genetic data. Twenty glass jars, each measuring 25 × 15 × 20 cm, were used for the experiment. Each jar was equipped with an independent pneumatic filtration device. The water volume was 6 liters, the temperature was maintained at 24 °C, and the TDS content of the water was approximately 150–200 mg/L. The daily duration of light was 8 h. The experiment lasted a total of 56 days. The choice of a 56-day duration was to align with the growth stage of the shrimp from juvenile to adult, allowing sufficient time to observe color development and pigment accumulation. During the experiment period, feed was provided twice a day at a rate of 0.03–0.05% of the shrimp’s body weight. Before each feeding, the bottom of the tank was cleaned to remove excess residual bait and excrement. The water was changed twice a week, with half of the tank water replaced each time.

#### 2.4.2 Hypoxia experiment

This experiment was conducted after the feeding experiment. For both body color and genetic data, 10 shrimp were used per tank, with three replicate sets for the genetic data. Twenty dissolved oxygen bottles, each with a volume of 300 mL, were used for the experiment. The temperature during the experiment was maintained at 24 °C. The dissolved oxygen concentration before the start of the hypoxia experiment was 6–7 mg/L. The hypoxic period lasted for 9 h and the bottles were sealed using a wax film (Bemis Company Inc., USA). At 0 and 9 h, dissolved oxygen values were measured and recorded using a dissolved oxygen meter. Body color in each treatment group was photographed, and genetic data were collected for subsequent analysis of body color and genetic information.

### 2.5 Body color data analysis

#### 2.5.1 Digital image capture and software-assisted measurement

ZEISS STEMI 305 EDU dissecting microscope (Carl Zeiss AG, Oberkochen, Germany) externally connected to the LeadView Full Color Cam digital imaging system (Leader Scientific Co., Ltd., NTPC, Taiwan) was used to photograph the shrimp body segments (S2 Fig). Adobe Photoshop CS5 12.0; Adobe Systems Inc., San Jose, CA, USA) was used for image analysis. A rectangular selection tool and an eye dropper tool were used with a fixed size of 51 × 51 pixels. The shrimp body color sampling area included body segments 3, 4, and 5 with three areas sampled for each body segment. The color parameters were calculated as the average of nine sampled areas (S3 Fig).

#### 2.5.2 CIELAB color space and color difference (ΔE*_ab_) value calculation

CIELAB color space is a color space defined by the International Commission on Illumination (Commission internationale de l’éclairage [CIE]). It includes three color parameters, L*, a*, and b*, which represent the lightness, redness, and yellowness, respectively [33]. The values of L*, a*, and b* are converted into L*, C* (chroma), and h* (hue angle) color spaces, respectively. The formulas for converting the C* and h* values are as follows: C*_ab_ = (a*^2^ + b*^2^)^1/2^ and h*_ab_ = tan^-1^(b*/a*), respectively.

The CIELAB color space, commonly abbreviated as CIELAB or LAB, was developed by the CIE in 1976 as a color-opponent space where three components (L*, a*, b*) are used to describe color in a three-dimensional space [33]. This model is widely utilized in industries such as printing, textiles, and graphic design, as well as in scientific research, because it is perceptually uniform—meaning that equal changes in values correspond to equal perceived changes in color by the human eye. This makes it ideal for color measurement, quality control, and assessing color differences in research and production environments.

Differences between colors were expressed through ΔE*_ab_ values defined by the CIE. ΔE*_ab_ was calculated using the latest revised version of CIEDE2000 [34], which provides the smallest error. ΔE*_ab_ value ≥ 2.3 is the just-noticeable difference that can be distinguished by the human eye [35].

### 2.6 Genetic data analysis

#### 2.6.1 Target gene primer design

Using the transcriptomes of freshwater shrimp previously established by our laboratory for gene screening, the genes related to carotenoid transmodal transport, binding, metabolism, and pigment cell transport distribution were selected. A total of 23 genes related to color development were selected for further analysis. Related genes included scavenger receptor class B member 1 (*SCARB1*), alcohol dehydrogenase (*ADH*), annexin (*ANX*), ATP-binding cassette (*ABC*), G protein-coupled receptor (*GPCR*), GTP cyclohydrolase (*GCH*), keratin (*KRT*), NADH-ubiquinone oxidoreductase (*NDU*), retinol dehydrogenase (*RDH*), solute carrier (*SLC*), xanthine dehydrogenase (*XDH*), crustacyanin-C1 (*CRCNC1*), cytochrome P450 (*CYP*), glutathione S-transferase (*GST*), crustacyanin-A2 (*CRCNA2*), red pigment-concentrating hormone (*RPCH*), vitellogenin (*VTG*), (very) low density lipoprotein receptor (*(V)LDLR*), flotillin (*FLOT*), beta-carotene oxygenase 1 (*BCO1*), beta-carotene oxygenase 2 (*BCO2*), neither inactivation nor afterpotential B (*NINAB*), and cytochrome b (*CYTB*) [36–38] (S1 Table). For genes related to antioxidant capacity and hypoxia response, five emergency-related genes were selected: catalase (*CAT*), manganese superoxide dismutase (*MnSOD*), Hypoxia-inducible factor (*HIF*), heat shock protein (*HSP*), and *GST* [39, 40] (S2 Table). Glyceraldehyde-3-phosphate dehydrogenase (*GAPDH*) was selected as the reference gene based on the literature recommendations for similar species [36, 37]. Geneious version 9.1.8 (Dotmatics, Boston, MA, USA) software was used to design the primers for the sequence-coding region.

#### 2.6.2 Total ribonucleic acid extraction and real-time quantification

To extract RNA from the shell and hepatopancreas tissues of cherry shrimp, the tissues of three individuals were pooled to ensure sufficient quantity for extraction. Total RNA was extracted using the EasyPure Total RNA Spin Kit (Bioman Scientific Co., Ltd., NTPC, Taiwan), and RNA reverse transcription was performed using the High-Capacity cDNA Reverse Transcription Kit (Applied Biosystems, Foster City, CA, USA). The sample cDNA concentration was diluted to 1 ng/μL, and the primer concentration was diluted to 10 μM. Amplification was performed using the Roche LightCycler 480 Real-Time Polymerase Chain Reaction (PCR) System (Roche Ltd., Basel, Switzerland). The real-time quantitative reaction provided the gene expression levels (Ct values) of the samples, which were calculated using the relative quantification (2^−ΔΔCt^) method [41].

### 2.7 Statistical analyses

IBM SPSS Statistics Version 22 (IBM, Armonk, NY, USA) was used for statistical analysis. Levene’s homogeneity test was performed before the analysis. For significance analysis of two groups of samples, an independent Sample *t*-test was used. For the significance analysis of more than two groups of samples, a one-way analysis of variance was used. A post hoc test was performed according to the number of samples (n). When the number of samples was equal, the least significant difference test was used, and when the number of samples was unequal, Tukey’s honest significant difference (HSD) test was used. Significance level was set at *p < .05, **p < .01, and ***p < .001.

## 3. Results

### 3.1 Effects of feed additives on the body color and related genes in cherry shrimp

#### 3.1.1 Analysis of the body color parameters of cherry shrimp

**Fig 1:**
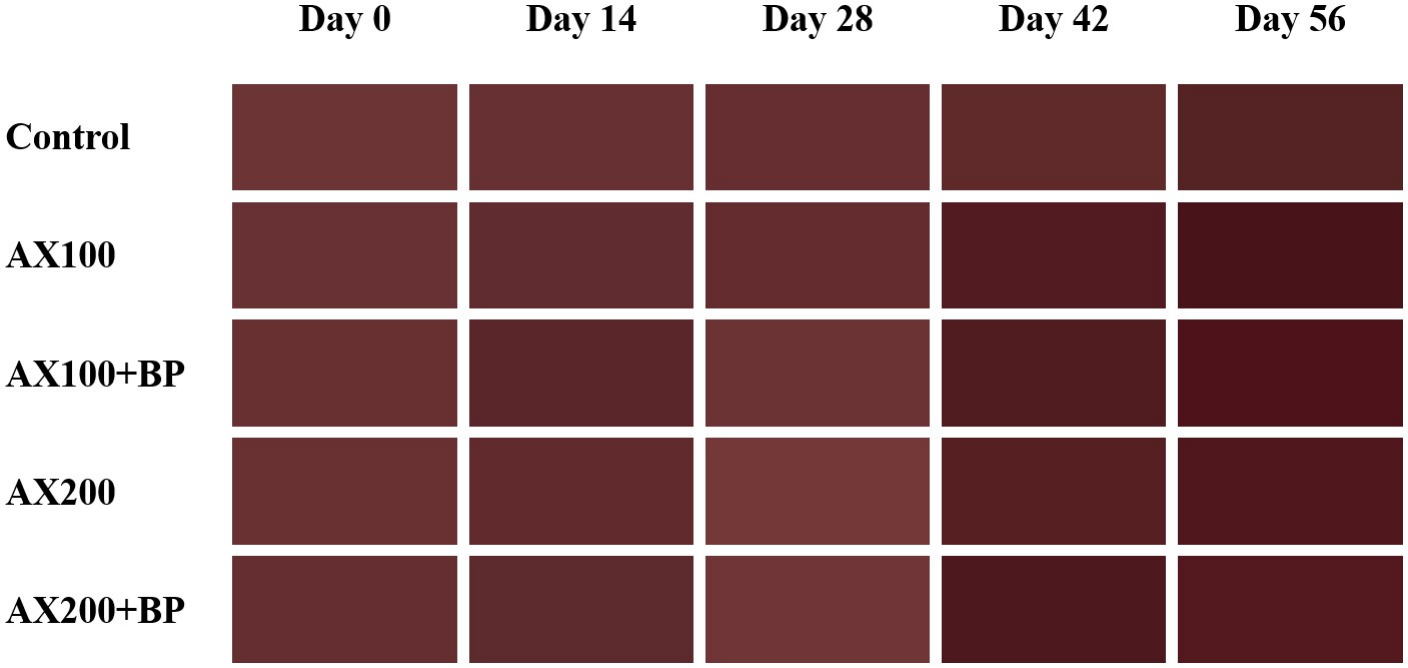
Changes in the body color of cherry shrimp (*N*. *davidi* var. red) across different feed treatment groups (Control, AX100, AX100 + BP, AX200, AX200 + BP) over time. Body color was recorded at five time points: 0, 14, 28, 42, and 56 days. The color parameters measured include *L*, a*, b*, C*, h*, and ΔE*_ab_ values, which indicate variations in lightness, redness, yellowness, chroma, hue angle, and overall color difference respectively.

We explored the impact of feed additives on body color by analyzing the cherry shrimp body color data. Cherry shrimps were divided into five groups, each receiving one of five different feed treatments: Control, AX100, AX100 + BP, AX200, and AX200 + BP groups. The experiment lasted for 56 d, with measurements taken at 14-d intervals (0, 14, 28, 42, and 56 d) to record the changes in body color. Various color parameters, including L*, a*, b*, C*, h*, and ΔE*_ab_ values, were measured (Fig 1).

Statistical analysis at 5% significance level revealed that, on day 0 of the experiment, the L*, a*, b*, C*, h*, and E *_ab_ values did not show significant differences among the feed treatment groups. On day 56 of the experiment, average L* values of the AX100 and AX100 + BP treatment groups were 15.56 ± 4.03 and 16.21 ± 2.69 respectively, which were significantly lower than the value (21.59 ± 1.43) of the Control treatment group (*p* < .05). The lower L* values indicated the darker body color of the shrimp.

Average a* values of the AX100 + BP and AX200 + BP treatment groups were 28.38 ± 1.63 and 27.73 ± 2.72, respectively, which were significantly higher than the value (24.37 ± 1.70) of the Control treatment group (*p* < .05). The higher a* values indicated that the shrimp had a more intense red body color. b* values did not show significant differences among the feed treatment groups, indicating that the b* value had no noticeable impact on the colour booster effect of the shrimp.

Average C* value of the AX100 + BP treatment group was 30.59 ± 1.73, which was significantly higher than the value (26.94 ± 2.03) of the Control treatment group (*p* < .05). The higher C* value indicated a more saturated body color of the shrimp. Average h* value of the AX100 treatment group was 21.35 ± 2.40, which was significantly lower than the value (25.19 ± 1.13) of the Control treatment group (*p* < .05). The lower h* value indicated a hue closer to red in the shrimp.

Average ΔE*_ab_ values of the AX100 and AX100 + BP treatments groups were 13.39 ± 2.31 and 14.23 ± 1.60, respectively, which were significantly lower than the value (16.06 ± 0.41) of the Control treatment group (*p* < .05). The lower ΔE*_ab_ value indicated that the shrimp body color was closer to the standard color of the Red Fire Shrimp (Table 2).

**Table 2:** Changes in the color parameters of cherry shrimp (*N*. *davidi* var. red) in different feed treatment groups^a^ over a 56-day period (mean ± SD, *n* = 20).

	Treatment				
	Control	AX100	AX100 + BP	AX200	AX200 + BP
Day 0					
L*	29.64 ± 6.99^a^	28.04 ± 5.06^a^	27.84 ± 4.03^a^	28.14 ± 7.32^a^	26.92 ± 6.70^a^
a*	25.52 ± 2.92^a^	25.24 ± 2.81^a^	25.74 ± 2.58^a^	25.69 ± 2.10^a^	25.65 ± 2.44^a^
b*	11.73 ± 1.61^a^	11.06 ± 1.32^a^	11.50 ± 1.38^a^	11.71 ± 1.84^a^	11.12 ± 1.44^a^
C*	28.14 ± 2.91^a^	27.58 ± 2.92^a^	28.22 ± 2.67^a^	28.27 ± 2.37^a^	27.99 ± 2.41^a^
h*	24.78 ± 3.42^a^	23.72 ± 2.28^a^	24.12 ± 2.45^a^	24.43 ± 3.00^a^	23.49 ± 3.02^a^
ΔE*_ab_	21.64 ± 4.60^a^	20.31 ± 3.47^a^	20.29 ± 2.54^a^	20.66 ± 4.93^a^	20.74 ± 6.15^a^
Day 14					
L*	27.65 ± 6.05^a^	25.52 ± 3.68^a^	22.88 ± 3.12^a^	25.35 ± 4.34^a^	24.51 ± 6.45^a^
a*	25.58 ± 2.11^a^	24.05 ± 3.31^a^	24.89 ± 1.44^a^	24.98 ± 2.43^a^	23.80 ± 2.65^a^
b*	10.67 ± 1.42^a^	9.48 ± 1.52^a^	9.95 ± 1.08^a^	11.13 ± 1.52^a^	9.92 ± 1.38^a^
C*	27.74 ± 2.22^a^	25.86 ± 3.53^a^	26.81 ± 1.63^a^	27.36 ± 2.68^a^	25.81 ± 2.75^a^
h*	22.64 ± 2.58^a^	21.51 ± 2.10^a^	21.76 ± 1.59^a^	24.00 ± 2.12^a^	22.67 ± 2.61^a^
ΔE*_ab_	20.12 ± 4.14^a^	18.22 ± 2.76^a^	16.80 ± 2.09^a^	18.48 ± 3.00^a^	17.73 ± 4.47^a^
Day 28					
L*	26.92 ± 6.47^a^	26.37 ± 4.46^a^	29.12 ± 3.85^a^	31.63 ± 6.39^a^	30.16 ± 7.07^a^
a*	26.02 ± 2.72^a^	26.70 ± 3.02^a^	26.16 ± 2.09^a^	26.74 ± 1.79^a^	26.10 ± 3.16^a^
b*	11.06 ± 1.16^a^	11.08 ± 1.69^a^	11.41 ± 1.60^a^	12.56 ± 1.92^a^	11.25 ± 1.73^a^
C*	28.29 ± 2.74^a^	28.94 ± 3.19^a^	28.55 ± 2.46^a^	29.58 ± 2.14^a^	28.46 ± 3.29^a^
h*	23.10 ± 2.31^a^	22.55 ± 2.67^a^	23.49 ± 1.90^a^	25.08 ± 2.93^a^	23.36 ± 2.95^a^
ΔE*_ab_	19.82 ± 4.13^a^	19.46 ± 3.02^a^	21.14 ± 2.75^a^	23.18 ± 4.53^a^	21.99 ± 5.28^a^
Day 42					
L*	25.02 ± 5.14^a^	19.12 ± 5.48^a^^b^	18.95 ± 4.07^a^^b^	20.90 ± 4.97^a^^b^	17.90 ± 4.99^b^
a*	24.78 ± 2.09^a^	26.51 ± 4.58^a^	25.53 ± 1.41^a^	25.44 ± 2.36^a^	25.83 ± 2.75^a^
b*	11.44 ± 1.68^a^	10.10 ± 1.72^a^	10.62 ± 1.12^a^	11.74 ± 1.97^a^	10.76 ± 1.48^a^
C*	27.32 ± 2.27^a^	28.46 ± 4.28^a^	27.66 ± 1.56^a^	28.05 ± 2.74^a^	28.01 ± 2.88^a^
h*	24.76 ± 3.07^a^	21.21 ± 4.47^a^	22.56 ± 1.87^a^	24.69 ± 2.82^a^	22.64 ± 2.55^a^
ΔE*_ab_	18.29 ± 3.55^a^	15.27 ± 3.09^a^^b^	14.84 ± 2.42^a^^b^	16.03 ± 3.26^a^^b^	14.48 ± 2.79^b^
Day 56					
L*	21.59 ± 1.43^a^	15.56 ± 4.03^b^	16.21 ± 2.69^b^	17.84 ± 5.22^a^^b^	18.79 ± 2.68^a^^b^
a*	24.37 ± 1.70^b^	26.48 ± 2.57^a^^b^	28.38 ± 1.63^a^	27.12 ± 2.26^a^^b^	27.73 ± 2.72^a^
b*	11.48 ± 1.23^a^	10.37 ± 1.61^a^	11.38 ± 0.95^a^	12.04 ± 1.91^a^	11.55 ± 0.83^a^
C*	26.94 ± 2.03^b^	28.46 ± 2.79^a^^b^	30.59 ± 1.73^a^	29.71 ± 2.51^a^^b^	30.05 ± 2.65^a^^b^
h*	25.19 ± 1.13^a^	21.35 ± 2.40^b^	21.85 ± 1.40^a^^b^	23.88 ± 3.02^a^^b^	22.72 ± 2.03^a^^b^
ΔE*_ab_	16.06 ± 0.41^a^	13.39 ± 2.31^b^	14.23 ± 1.60^b^	14.92 ± 2.93^a^^b^	15.33 ± 1.66^a^^b^

^a^ AX100—Astaxanthin 100 mg/kg, BP—*Bidens pilosa*.

^b^ Different letters indicate significant differences within the same row (p < 0.05).

**Fig 2:**
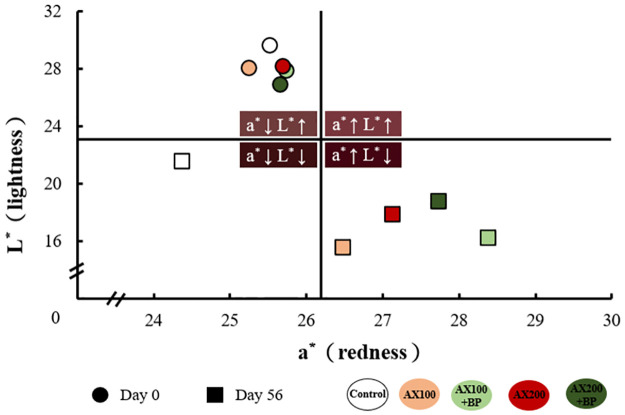
Two-dimensional quadrant analysis of cherry shrimp (*N*. *davidi* var. red) bodycolor parameters (L* and a* values) in different feed treatment groups on days 0 and 56. Data points represent mean values ± standard deviation (*n* = 20).

If the L* and a* values (lightness and redness) were analyzed on days 0 and 56 of the experiment using the two-dimensional quadrant method. On day 0, each feed-treatment group was distributed in the second quadrant. Body color showed a higher L* value and lower a* value, indicating a lighter and less saturated color. As the feeding period progressed, by day 56, the distribution of the treatment groups in the quadrants varied. The Control treatment group was distributed in the third quadrant, with body color showing a significantly reduced L* value, leading to a deeper and more intense color. In contrast, the AX100, AX100 + BP, AX200, and AX200 + BP treatment groups were all distributed in the fourth quadrant, showing significantly reduced L* values and significantly increased a* values, resulting in a fuller, deeper, and more vibrant red color, reflecting greater pigment accumulation (Fig 2).

#### 3.1.2 Analysis of color development-related genes in cherry shrimp

**Fig 3:**
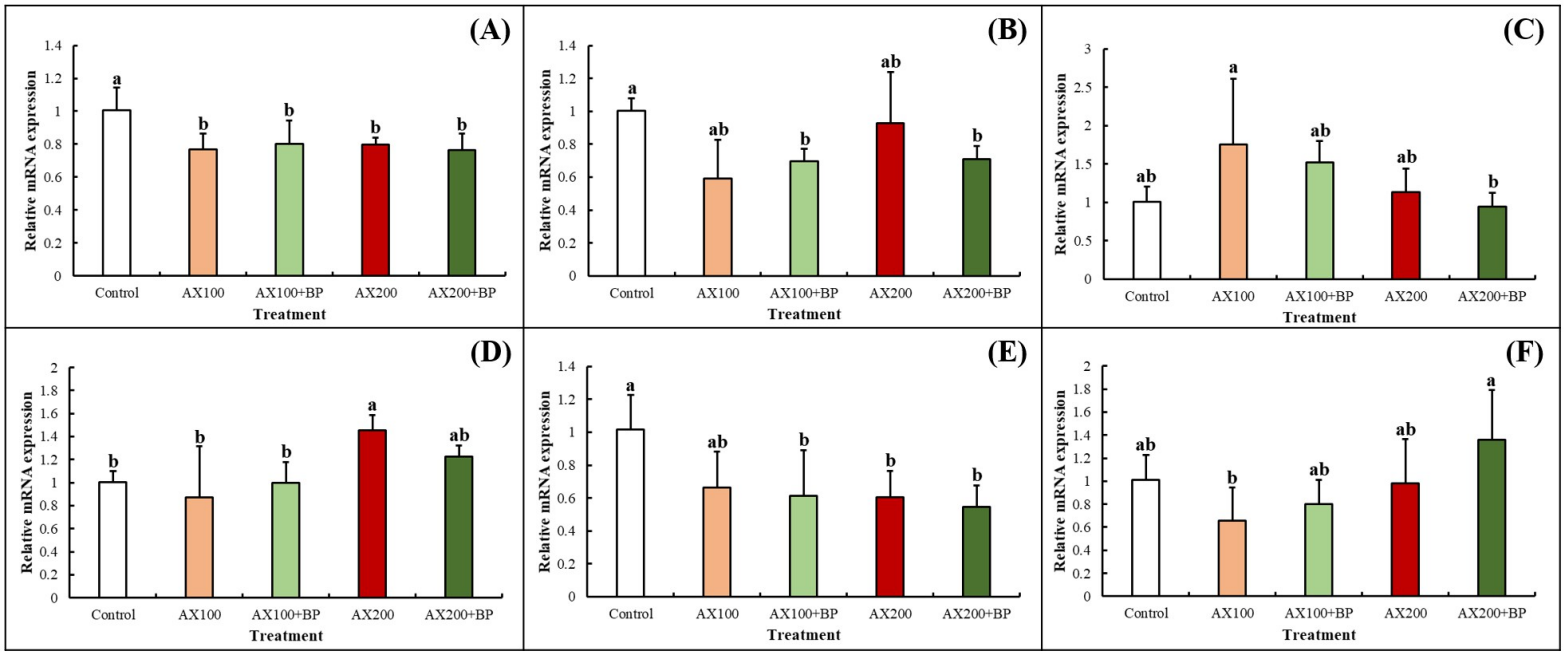
Expression analysis of color-related genes in the hepatopancreas and shell tissues of cherry shrimp (*N*. *davidi* var. red) fed with vehicle, astaxanthin alone and in combination with *Bidens pilosa* for 56 days. RT-qPCR was conducted to measure the expression level of *ABCA5* (A), *RDH11* (B), *SLC28A3* (C), *CRCNA2* (D), *GSTM2* (E), and *CYP18A1* (F). The relative expression level of the indicated genes was nromalized to the reference gene, Glyceraldehyde 3-phosphate dehydrogenase (GAPDH).

Genetic data analysis was conducted to explore the effects of feed additives on the expression levels of color-related genes in the hepatopancreatic tissues of cherry shrimp. On day 56, samples from the hepatopancreas and shell tissues were extracted for reverse transcription-quantitative PCR (RT-qPCR). Based on the calculation of relative performance, the control treatment group was used as a benchmark to compare the performance differences among the different feed treatment groups. Statistical analysis revealed that, at the 5% significance level, three groups of genes (*ABCA5*, *RDH11*, and *SLC28A3*) exhibited significant differences in the hepatopancreatic tissues of different feed treatment groups. Additionally, three groups of genes (*CRCNA2*, *GSTM2*, and *CYP18A1*) exhibited significant differences in the shell tissues of different feed treatment groups (Fig 3).

### 3.2 Effects of feed additives on the body color stability and related genes in cherry shrimp under hypoxic stress

#### 3.2.1 Analysis of ΔE*_ab_ values before and after hypoxic stress in cherry shrimp

At 0 and 9 h of the hypoxic stress experiment, body color changes were photographed and recorded to measure the following color parameters in each feed treatment group: L*, a*, and b* values. This enabled the calculation of the ΔE*_ab_ values before and after hypoxia for each feed treatment group. Statistical analysis at the 5% significance level revealed the average ΔE*_ab_ values before and after hypoxia stress in the AX100 + BP and AX200 + BP treatment groups as 1.64 ± 0.70 and 1.81 ± 1.22, respectively. These values were significantly lower than the value (3.29 ± 1.51) observed in the Control treatment group (p < .05) and were all below the minimum color difference value of 2.3 that can be distinguished by the human eye. This indicated significant differences in the body color changes before and after hypoxic stress that were not discernible by the human eye (Table 3).

**Table 3:** Analysis of color parameters (*L*, *a*, b*) and color difference (ΔE*_ab_) values^a^ before and after hypoxic stress in cherry shrimp (*N*. *davidi* var. red) under different treatment conditions (*n* = 10).

	Treatment				
	Control	AX100	AX100 + BP	AX200	AX200 + BP
**Before**					
L*	24.36 ± 5.63	17.31 ± 4.48	16.18 ± 2.83	14.68 ± 4.81	16.50 ± 3.36
a*	27.62 ± 2.83	27.22 ± 2.15	28.48 ± 1.69	26.86 ± 2.07	27.96 ± 2.44
b*	13.27 ± 1.87	10.99 ± 1.52	11.33 ± 0.98	12.08 ± 1.98	11.52 ± 0.84
**After**					
L*	20.82 ± 7.17	16.48 ±5.97	16.09 ± 4.33	17.68 ± 4.94	16.90 ± 4.44
a*	26.52 ± 2.70	26.93 ± 2.71	26.43 ± 2.02	26.16 ± 1.92	27.90 ± 2.65
b*	11.47 ± 2.00	10.90 ± 2.16	10.67 ± 1.17	11.22 ± 1.43	11.64 ± 1.25
ΔE*_ab_	3.29 ± 1.51^a^	2.25 ± 1.02^a^^b^	1.64 ± 0.70^b^	2.41 ± 1.10^a^^b^	1.82 ± 1.22^b^

^a^ Values are mean ± standard deviation (*n* = 10).

^b^ Different superscripts within the same row indicate statistically significant differences (p < 0.05).

#### 3.2.2 Analysis of stress-related gene expression in cherry shrimp

**Fig 4:**
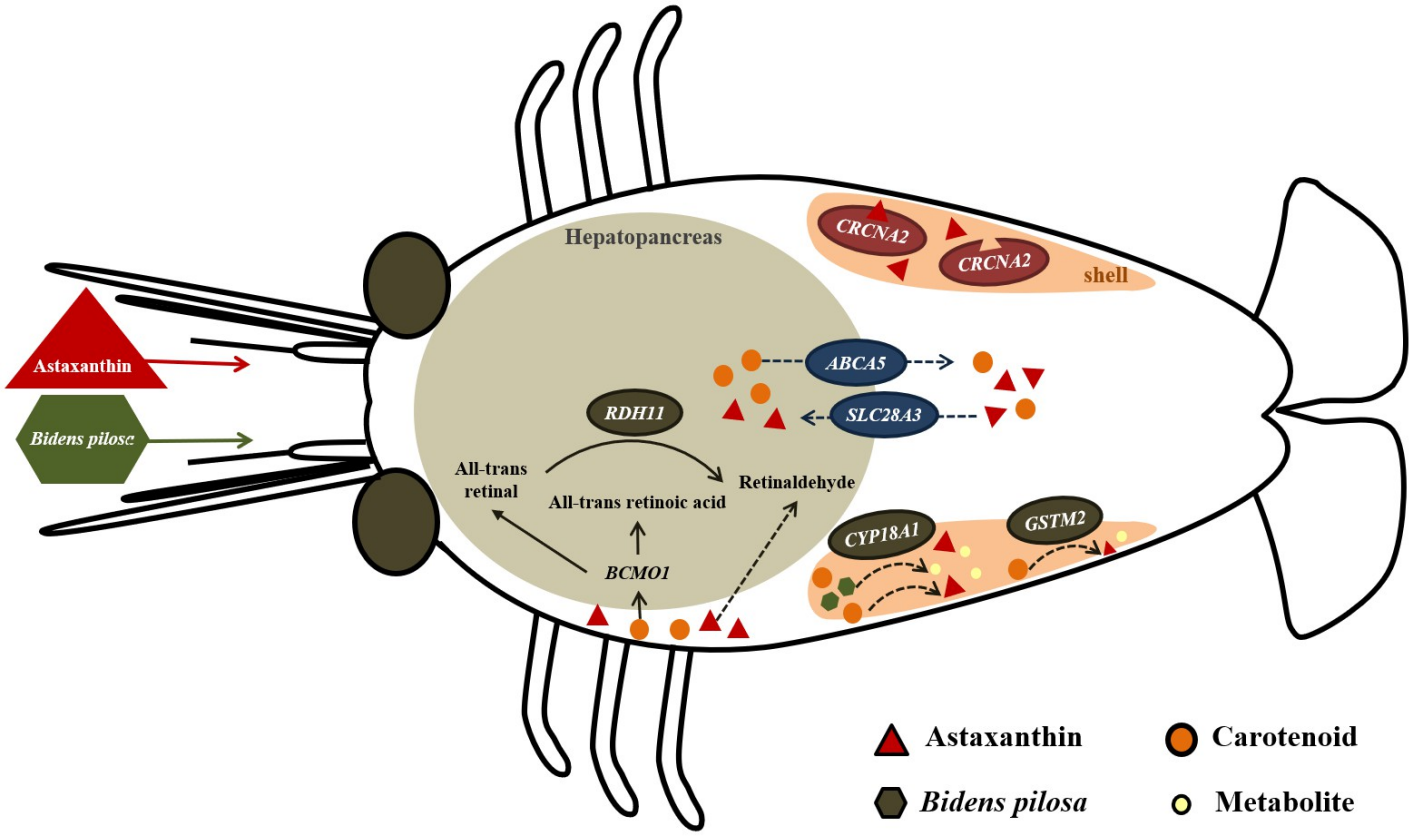
Analysis of stress-related gene expression in cherry shrimp (*N*. *davidi* var. red). (A) Differences in expression levels of all feed treatment groups before and after hypoxia. (B) Expression levels of gene (*CAT*) in all feed treatment groups after hypoxia. Data are represented as the mean ± standard deviation (n = 3). Means with different letters (a, b) are significantly different (p < .05). Numerical value indicates the relative performance amount (2^−ΔΔCt^) calculated using the control group as the reference point. Glyceraldehyde 3-phosphate dehydrogenase (GAPDH) was used as the reference gene for expression calculations.

At 0 and 9 h of the hypoxic stress experiment, samples of hepatopancreatic tissue were extracted, and five sets of stress-related gene primers were used for RT-qPCR. Based on the calculation of relative expression levels, the control treatment group was used as the benchmark to compare the gene expression differences among the feed treatment groups. Statistical analysis showed that, at the 5% significance level, one group of genes (*CAT*) showed significant differences among the feed treatment groups (Fig 4).

## 4. Discussion

### 4.1 Effects of feed additives on the color of ornamental and edible aquatic species

Several studies have proposed a correlation between the carotenoid content in organisms and body color parameters. Typically, as the carotenoid content of an organism increases, its L* value decreases, indicating a negative correlation between the two parameters [42]. As the feeding time increases, carotenoids in the feed act as a color booster in organisms, altering various color parameters. This typically results in lower L* values, higher a* values, and increased or unchanged b* values. Consequently, the C* value increases, making the body color appear darker [43–45]. A lower h* value indicates a redder body color, whereas a higher h* value indicates a more yellowish color. By adding carotenoids to the feed, the h* value can be reduced, making the body color closer to red [46].

This study explored the color-boosting effect of feed additives on cherry shrimp body color. After 56 d of feeding, various color parameters were measured, and the results were consistent with the trends observed in the literature. The experiment results showed that dietary supplementation with either 100 mg/kg astaxanthin (AX100) alone or in combination with 5 g/kg *B*. *pilosa* (AX100 + BP) resulted in significantly lower ΔE*_ab_ values, indicating that the body color was closest to that of the standard product. This included a reduction in lightness, a decrease in color hue angle with AX100, and increases in redness and chroma with AX100+BP. These two treatment groups were more effective than the AX200 and AX200 + BP groups treated with 200 mg/kg astaxanthin. Dananjaya *et al*. (2017) reported that the enhancement of body color is not always directly proportional to the amount of astaxanthin administered. They suggested that this phenomenon may result from limitations in the uptake, transport, and accumulation of astaxanthin, leading to saturation effects in pigment deposition [47].

Analysis of ΔE*_ab_ values showed that *Bidens pilosa* had no significant impact on body color, as AX100 and AX100+BP groups exhibited similar results, and AX200 and AX200+BP groups demonstrated consistent values. This indicates that the amount of astaxanthin, rather than *Bidens pilosa*, was the key factor enhancing body color. Current studies suggest that both astaxanthin and *Bidens pilosa* are safe additives within appropriate dosage ranges, as they have not demonstrated toxicity or adverse effects in various species [21, 27]. No side effects were observed in this study, but further research is needed to confirm the absence of long-term risks.

### 4.2 Effects of feed additives on color development-related genes in crustaceans

RT-qPCR revealed significant differences in the expression of the six color genes between the different feed treatment groups. TRINITY_DN17444 was annotated as the ATP-binding cassette subfamily A (ABC1) member 5 (*ABCA5*) gene. Abcg2-1a protein levels in the intestine were 2.5-times higher in the light-red individuals than in the dark-red individuals of Atlantic salmon. Abcg2-1a limits the availability of astaxanthin in the muscles by transferring it from enterocytes to the intestinal lumen, thereby reducing muscle pigment accumulation [48]. After 56 d of feeding, *ABCA5* gene expression in AX100, AX 100+BP, AX200, and AX200+BP groups was significantly lower than that in the control treatment group. This may be because the addition of the red pigment leads to an increase in extracellular carotenoid content and reduces the transport function of the *ABCA5* gene, thereby increasing carotenoid accumulation in the hepatopancreatic tissue.

TRINITY_DN2480 was annotated as the *RDH11* (retinol dehydrogenase 11) gene. *bco2* levels in intestinal cells are lower in dark-red individuals than in the light-red individuals of *Oncorhynchus tshawytscha*. This indicates that light-red individuals convert astaxanthin into retinol through β-carotene oxygenase and then into retinaldehyde through retinol dehydrogenase, whereas dark-red individuals mainly produce vitamin A by directly decomposing astaxanthin [49]. An increase in retinol-related enzymes reduces the utilization of astaxanthin and accelerates the metabolism of carotenoids into colorless derivatives, causing differences in body color expression [50]. Studies on resistance to traditional chemotherapy in human cancer treatment have found that certain Chinese herbs and spices can modulate retinol-related enzymes and inhibit the activity of retinol-related metabolites (RALDHs) [51]. Another study on peptic ulcers in rats indicated that an oral Chinese herbal formula (Sulongga-4, SL-4) could downregulate the expression of retinol metabolism-related genes in the gastroduodenal tissue [52]. In this experiment, after 56 d of feeding, *RDH11* gene expression in AX 100+BP and AX200+BP groups was significantly lower than that in the control treatment group. This effect may be attributed to the increased astaxanthin content from supplementation, which leads to the direct breakdown of astaxanthin through the primary vitamin A production pathway. This direct conversion is more efficient than the indirect breakdown of retinol, resulting in reduced *RDH11* gene expression. Consequently, more astaxanthin is stored in the cells for later use, which aligns with findings reported in previous studies. In addition, research on Chinese herbal medicine has found that these medicines can inhibit the expression of retinol-related enzymes. Therefore, in the treatment group with the combined addition of astaxanthin and *B*. *pilosa*, this regulatory effect may further reduce the expression of the *RDH11* gene.

TRINITY_DN4076 was annotated as *SLC28A3* gene. The correlation between the *SLC* gene family and melanin synthesis and carotenoid transport has been reported in *Euthynnus affinis* [53], Malaysian red tilapia [54], Nile tilapia (*Oreochromis niloticus*) [55], *Tetranychus urticae* [56], and *Neocaridina denticulata* [37]. However, no study has investigated the correlation between the SLC28 gene family and pigments. After 56 d of feeding, the *SLC28A3* gene expression in AX100 group was significantly higher than that in the AX200+BP treatment group. This may be due to the addition of astaxanthin, which alters carotenoid content and increases carotenoid accumulation in the hepatopancreas, leading to changes in gene expression.

TRINITY_DN49 was annotated as *CRCNA2* gene. *CRCN* levels are lower in the orange-red individuals than in the wild-type individuals of *Exopalaemon carinicauda*, potentially affecting the release of free astaxanthin in this species [57]. Many studies have used RNA interference for CRCN-related genes in shrimp species such as *Penaeus monodon* [58], *Exopalaemon carinicauda* [59], and *Macrobrachium rosenbergii* [60]. These studies yielded similar results, such as increased redness in color parameters or a change in body color to orange-red, whereas the blue subcutaneous pigment was significantly reduced. After 56 d of feeding, the *CRCNA2* gene expression in the Control, AX100, and AX100 + BP groups was significantly lower than that in the AX200 treatment group. This may be because the addition of excessive astaxanthin increased the shrimp content of red pigment-binding protein and reduced the amount of free astaxanthin, thereby reducing the redness of the body color. Therefore, the body color observed in the AX200 and AX200 + BP treatment groups was not as favorable as that observed in the AX100 and AX100 + BP treatment groups. In the Control treatment group, the astaxanthin content in the feed was lower compared to the AX100 and AX100 + BP treatment groups. This lower astaxanthin availability led to a less vibrant body color in the Control group, despite similar levels of *CRCNA2* gene expression across Control, AX100 and AX100+BP groups.

TRINITY_DN5642 was annotated as the *GSTM2* (glutathione S-transferase Mu 2) gene. *GSTA2* is involved in the binding and metabolism of carotenoids and affects dichromatism in bird feathers [61]. In this experiment, after 56 d of feeding, the expression of the *GSTM2* gene in AX 100+BP, AX200, and AX200+BP groups was significantly lower than that in the control treatment group. This may be because the addition of astaxanthin increased the metabolite content, thereby reducing protein binding and metabolic efficiency.

TRINITY_DN56342 was annotated as the cytochrome P450 family 18 subfamily A member 1 (*CYP18A1*) gene. Research on red pigment production in birds has indicated that in the skin and liver tissues of black-headed red goldfinch (*Spinus cucullata*), cytochrome P450 (*CYP2J19*) gene expression is significantly higher than that in canaries (*Serinus canaria*). This gene catalyzes the conversion of yellow carotenoids into red ketone carotenoid pigments and participates in retinal carotenoids in the retina [62, 63]. In a study on *Tetranychus urticae*, the expression of the cytochrome P450 (*CYP389B1*) gene was upregulated in female red individuals, and RNA interference with the mRNA of the gene resulted in a reduction in red pigment, indicating that this gene regulates orange-red body color [56]. Cytochrome P450 plays a key role in the metabolism of hydrophobic endogenous substrates and ingested exogenous compounds including flavonoids [64]. After 56 d of feeding, the expression level of *CYP18A1* gene in the AX100 group was significantly lower than that in the AX200+BP treatment group. This result can be discussed in terms of two aspects: the addition of astaxanthin to the feed, and the compound addition of *B*. *pilosa*. From the above studies suggested that the cytochrome P450 (CYP) gene can be converted into astaxanthin. It is speculated that the four treatment groups with added astaxanthin had higher or saturated astaxanthin content, thereby inhibiting the expression of the *CYP18A1* gene. In the AX100 + BP and AX200 + BP treatment groups, the expression of the *CYP18A1* gene was higher than that in the single astaxanthin treatment groups. This may be due to the addition of *B*. *pilosa* to the feed, leading to an increase in the flavonoid content of the shrimp body, thereby promoting the metabolism of this gene and upregulating the expression of the *CYP18A1* gene.

**Fig 5:**
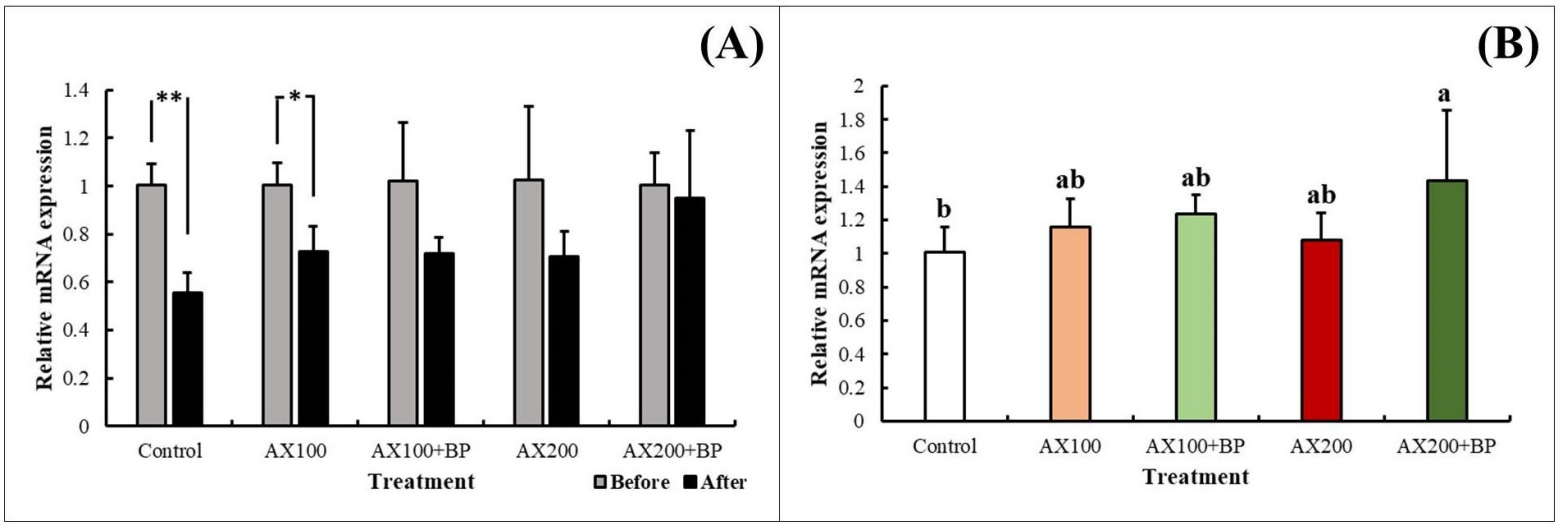
Correlations between feed additives and color-related genes. Crustacyanin-A2 (*CRCNA2*; TRINITY_DN49) encodes a protein associated with carotenoid binding, ATP-binding cassette subfamily A member 5 (*ABCA5*; TRINITY_DN17444) and solute carrier family 28 member 3 (*SLC28A3*; TRINITY_DN4076) are associated with carotenoid transport across membranes, and retinol dehydrogenase 11 (*RDH11*; TRINITY_DN2480), glutathione S-transferase mu 2 (*GSTM2*; TRINITY_DN5642), and cytochrome P450 family 18 subfamily A member 1 (*CYP18A1*; TRINITY_DN56342) are associated with carotenoid metabolism. Data are represented as the mean ± standard deviation (*n* = 3). Means with different letters are significantly different (p < .05). Numerical value indicates the relative performance (2^−ΔΔCt^) calculated using the control group as the reference point. Glyceraldehyde 3-phosphate dehydrogenase (GAPDH) was used as the reference gene for expression calculations.

These results revealed significant differences in the mRNA levels of the six sets of genes among different feed treatment groups, indicating a correlation between feed additives and color-related genes (Fig 5). Specifically, the genes *ABCA5* (TRINITY_DN17444), *SLC28A3* (TRINITY_DN4076), *CRCNA2* (TRINITY_DN49), and *GSTM2* (TRINITY_DN5642) are related to astaxanthin, showing significant differences between the feed addition treatment groups (AX100, AX100+BP, AX200, and AX200+BP) and the control group. In contrast, *RDH11* (TRINITY_DN2480) and *CYP18A1* (TRINITY_DN56342) are linked to both astaxanthin and *B*. *pilosa*, showing significant differences between the feed combined addition of astaxanthin and *B*. *pilosa* (AX100+BP and AX200+BP) and other treatment groups.

### 4.3 Effects of feed additives on body color changes under emergency conditions

Fish body color changes in stressful environments, such as high density, high temperature, and confined environments. These changes have been reported in red sea bream (*Pagrus pagrus*) [65], red tilapia (*Oreochromis* spp.) [66], and golden sea bream (*Chrysophrys auratus*) [67]. Additionally, treatment with anesthetics or feed additives before stress affects the fish color in killifish (*Girardinus metallicus*) [68] and flounder (*Paralichthys olivaceus*) [69]. One study reported that adding *Arthrospira platensis* to the feed of yellow catfish (*Pelteobagrus fulvidraco*) improves its body color under oxidative stress caused by air exposure. The group treated with *Arthrospira platensis* showed clearer black spots and more saturated yellow color before and after air exposure, suggesting that antioxidant components, such as lutein, in algae combat oxidative stress-induced color changes [14].

Here, analysis of ΔE*_ab_ values in each treatment group before and after hypoxia revealed that the changes in body color were lower in the astaxanthin treatment groups (AX100, AX200, AX100 + BP, and AX200+BP) than in the Control group, indicating that adding astaxanthin to the feed stabilizes the body color in stressful environments. In AX100 + BP and AX200 + BP treatment groups compounded with *B*. *pilosa*, the ΔE*_ab_ values were significantly lower than that in the Control group, indicating that *B*. *pilosa* significantly affects the body color stability and exerts synergistic effects with astaxanthin.

### 4.4 Emergency-related genes

Here, RT-qPCR revealed significant differences in one set of emergency-related genes in different feed treatment groups. TRINITY_DN1624 is annotated as a *CAT* (catalase) gene. Many studies have reported *CAT* downregulation in *Oncorhynchus mykiss* [12] and Pacific white shrimp (*Litopenaeus vannamei*) [70] in stressful environments. Feed additives also affect the gene activity. Addition of Chinese herbal medicine to the feed improves the CAT activity in largemouth bass (*Micropterus salmoides*) [71], Pacific white shrimp (*Litopenaeus vannamei*) [72], and Nile tilapia (*Oreochromis niloticus*) [73]. Addition of astaxanthin under acute hypoxia or air exposure significantly increases *CAT* levels in *Trachinotus ovatus* [74] and *Pelteobagrus fulvidraco* [14].

In this experiment, after 9 h of hypoxic stress, *CAT* gene expression in the different feed treatment groups was lower than that before hypoxia, with the most significant decrease observed in the control group. After hypoxia, in the AX200 + BP treatment group, which included astaxanthin and *B*. *pilosa*, the *CAT* gene expression levels were significantly higher than that in the control group. This may be due to the addition of astaxanthin and *B*. *pilosa* to the feed, which increased the antioxidant content, thereby reducing the utilization of the *CAT* gene and resulting in higher *CAT* gene expression. This finding is consistent with trends reported in the literature.

Based on the previous discussion, three possible mechanisms are summarized to explain how the combination of astaxanthin and *B*. *pilosa* compounds synergistically enhances the ability of ornamental shrimp to recover from environmental stress. These mechanisms include: (1) Enhanced ROS scavenging and antioxidant defense: The combined effects of astaxanthin and *B*. *pilosa* compounds provide a more comprehensive antioxidant defense. The lipid-soluble nature of astaxanthin complements the water-soluble antioxidant compounds in BP, offering a broad spectrum of reactive oxygen species (ROS) scavenging effects across different cellular compartments [29, 75]. (2) Cooperative Activation of the Nrf2 Pathway: Both astaxanthin and *B*. *pilosa* compounds can activate the Nrf2 pathway, leading to the upregulation of various antioxidant and cytoprotective genes. This synergistic activation ensures a robust and sustained response to oxidative stress. Nrf2 regulates the basal and inducible expression of a series of antioxidant response element-dependent genes, thereby controlling the physiological and pathophysiological outcomes of oxidative stress exposure [76, 77]. (3) Reduction of Oxidative and Inflammatory Damage: The anti-inflammatory properties of *B*. *pilosa* compounds, combined with the antioxidant effects of astaxanthin, help reduce oxidative damage and inflammation. This dual action mitigates the adverse effects of environmental stressors and promotes recovery and resilience [21, 78].

Recent studies have provided detailed insights into the digestive system of *Neocaridina heteropoda* [79] and examined the influence of microbial interactions on the efficacy of feed additives [80]. These findings are highly valuable for guiding future research directions. To further enhance the health and color quality of ornamental shrimp, it is crucial to continue exploring the dietary effects of supplements such as astaxanthin and *Bidens pilosa*. Understanding how these supplements are absorbed and metabolized within the shrimp’s digestive system, as well as their interaction with the shrimp’s intestinal microbiota, will provide deeper insights into their overall efficacy [81]. These interactions can significantly impact shrimp growth, body coloration, and stress tolerance. Future research should focus on quantitative trait loci (QTL) mapping and nutrigenomics to elucidate the genetic mechanisms underlying these traits. Such molecular breeding approaches can lead to the development of high-quality shrimp strains with enhanced commercial value [36, 82, 83]. By integrating whole-genome analysis and nutrigenomic techniques [84, 85], researchers can identify specific genetic markers associated with desirable traits, paving the way for more sustainable aquaculture practices.

## 5. Conclusion

Here, addition of different concentrations of astaxanthin and *B*. *pilosa* extract to the feed significantly enhanced the cherry shrimp color expression and stability under hypoxic stress during transportation. Our results revealed the combination of 100 mg/kg astaxanthin and 5 g/kg *B*. *pilosa* extract (AX100 + BP) as the optimal feed additive for enhanced performance of color-related genes. Our findings suggest that the effects of feed additives on color may be related to gene regulation mechanisms. Overall, this study recommends the use of feed additives containing astaxanthin and *B*. *pilosa* extract during cultivation and transportation to enhance the color parameters and hypoxia tolerance of cherry shrimp.

## Supporting information

S1 Fig Appearance and body color of *N*. *davidi* var. red. “Reprinted from [ref] under a CC BY license, with permission from [name of publisher], original copyright [original copyright year].” (A) Red cherry shrimp. (B) Red fire shrimp. This figure illustrates the physical appearance and body color of cherry shrimp used in the study. Image (A) shows a Red cherry shrimp, while image (B) depicts a Red fire shrimp. These images help visualize the differences in color intensity and body morphology between different treatments.
(TIF)

S2 Fig Procedure to capture digital images of the body color of ornamental shrimp. “Reprinted from [ref] under a CC BY license, with permission from [name of publisher], original copyright [original copyright year].” This figures shows the step-by-step procedure used to obtain the images for body color analysis of cherry shrimp in this study. Accuracy and consistency of image capture are critical to assess the changes in shrimp body color and evaluate the impacts of dietary supplementation with astaxanthin and *Bidens pilosa* under both normal and hypoxic conditions.
(TIF)

S3 Fig Color data collection points on the body of ornamental shrimp. “Reprinted from [ref] under a CC BY license, with permission from [name of publisher], original copyright [original copyright year].” (1) Circles denote the different segments of the shrimp body. (2) Square numbers indicate the sampling areas. (3) Yellow box indicates the sampling size (51 × 51 pixels) and quantity (3 per segment).
(TIF)

S1 Table Annotation database for color development-related genes in the Neocaridina shrimp transcriptome. (DOCX)

S2 Table Annotation database for emergency -related genes in the Neocaridina shrimp transcriptome. (DOCX)
