# Supplementary figures and images for: Enhancing the color and stress tolerance of cherry shrimp (Neocaridina davidi var. red) using astaxanthin and Bidens Pilosa
Source: PLoS One. 2024 Dec 19;19(12):e0315585. doi: 10.1371/journal.pone.0315585 (PMC11658619; doi:10.1371/journal.pone.0315585)

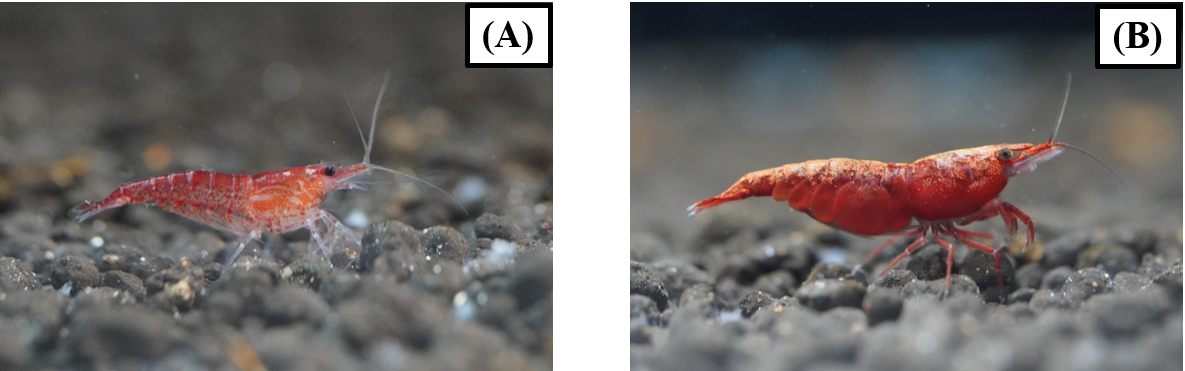

Supplement: S1 Fig — “Reprinted from [ref] under a CC BY license, with permission from [name of publisher], original copyright [original copyright year].” (A) Red cherry shrimp. (B) Red fire shrimp. This figure illustrates the physical appearance and body color of cherry shrimp used in the study. Image (A) shows a Red cherry shrimp, while image (B) depicts a Red fire shrimp. These images help visualize the differences in color intensity and body morphology between different treatments. (TIF) [file pone.0315585.s001.tif]

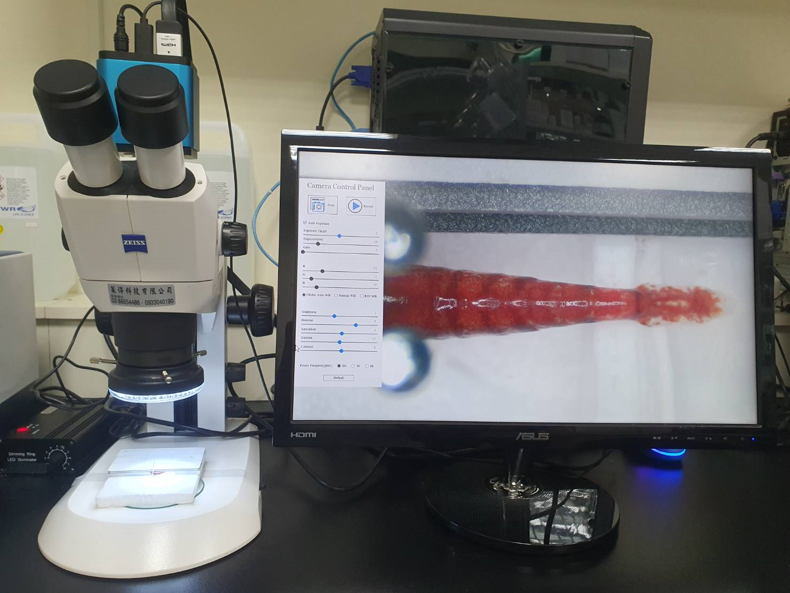

Supplement: S2 Fig — “Reprinted from [ref] under a CC BY license, with permission from [name of publisher], original copyright [original copyright year].” This figures shows the step-by-step procedure used to obtain the images for body color analysis of cherry shrimp in this study. Accuracy and consistency of image capture are critical to assess the changes in shrimp body color and evaluate the impacts of dietary supplementation with astaxanthin and Bidens pilosa under both normal and hypoxic conditions. (TIF) [file pone.0315585.s002.tif]

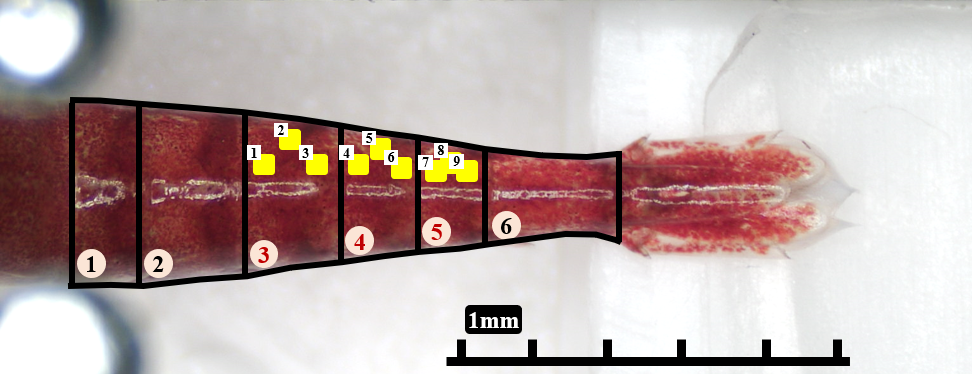

Supplement: S3 Fig — “Reprinted from [ref] under a CC BY license, with permission from [name of publisher], original copyright [original copyright year].” (1) Circles denote the different segments of the shrimp body. (2) Square numbers indicate the sampling areas. (3) Yellow box indicates the sampling size (51 × 51 pixels) and quantity (3 per segment). (TIF) [file pone.0315585.s003.tif]
