# Supplementary material for: Enhancing the color and stress tolerance of cherry shrimp (Neocaridina davidi var. red) using astaxanthin and Bidens Pilosa
Source: PLoS One. 2024 Dec 19;19(12):e0315585. doi: 10.1371/journal.pone.0315585 (PMC11658619; doi:10.1371/journal.pone.0315585)
Supplement: S1 Table — (DOCX) [file pone.0315585.s004.docx]

**S1 Table. Annotation database for color development-related genes in the Neocaridina shrimp transcriptome.**

| **Transcript_ID** | **Pfam** | **Swiss – Prot** | **NCBI BLAST** |
| --- | --- | --- | --- |
| TRINITY_DN1169_c0_g1_i6 | Alcohol dehydrogenase GroES-like domain | ADHX_SPAAU Alcohol dehydrogenase class-3 OS=*Sparus aurata* | PREDICTED: *Portunus trituberculatus* alcohol dehydrogenase class-3-like (LOC123520515), mRNA |
| TRINITY_DN40593_c0_g1_i2 | Annexin | ANXA7_BOVIN Annexin A7 OS=*Bos taurus* | - |
| TRINITY_DN17444_c1_g2_i6 | ABC-2 family transporter protein | ABCA5_HUMAN ATP-binding cassette sub-family A member 5 OS=*Homo sapiens* | PREDICTED: *Procambarus clarkii* cholesterol transporter ABCA5-like (LOC123773774), transcript variant X4, mRNA |
| TRINITY_DN14056_c0_g1_i1 | Rhodopsin-like GPCR transmembrane domain | GP107_RAT Protein GPR107 OS=*Rattus norvegicus* | PREDICTED: *Cherax quadricarinatus* protein GPR107-like (LOC128693830), mRNA |
| TRINITY_DN19599_c0_g1_i1 | GTP cyclohydrolase I | GCH1_DROME GTP cyclohydrolase 1 OS=*Drosophila melanogaster* | PREDICTED: *Penaeus chinensis* GTP cyclohydrolase 1-like (LOC125031760), transcript variant X2, mRNA |
| TRINITY_DN652_c0_g1_i1 | Keratinocyte-associated protein 2 | KTAP2_AEDAE Protein KRTCAP2 homolog OS=*Aedes aegypti* | - |
| TRINITY_DN12772_c0_g1_i1 | NADH-ubiquinone oxidoreductase B18 subunit (NDUFB7) | NDUB7_BOVIN NADH dehydrogenase [ubiquinone] 1 beta subcomplex subunit 7 OS=*Bos taurus* | PREDICTED: *Diachasma alloeum* NADH dehydrogenase [ubiquinone] 1 beta subcomplex subunit 7 (LOC107041048), mRNA |
| TRINITY_DN2480_c0_g1_i4 | short chain dehydrogenase | RDH11_MOUSE Retinol dehydrogenase 11 OS=*Mus musculus* | - |
| TRINITY_DN4076_c0_g1_i2 | Na^+^ dependent nucleoside transporter C-terminus | S28A3_RAT Solute carrier family 28 member 3 OS=*Rattus norvegicus* | PREDICTED: *Anarrhichthys ocellatus* solute carrier family 28 member 1 (slc28a1), transcript variant X2, mRNA |
| TRINITY_DN1053_c0_g1_i2 | Aldehyde oxidase and xanthine dehydrogenase, a/b hammerhead domain | XDH_DROSU Xanthine dehydrogenase OS=*Drosophila subobscura* | - |
| TRINITY_DN2115_c0_g1_i1 | Lipocalin / cytosolic fatty-acid binding protein family | CRC1_HOMGA Crustacyanin-C1 subunit OS=*Homarus gammarus* | - |
| TRINITY_DN56342_c0_g1_i1 | Cytochrome P450 | CP18A_DROME Cytochrome P450 18a1 OS=*Drosophila melanogaster* | *Neocaridina denticulata* CYP18A1 mRNA, partial cds |
| TRINITY_DN5642_c1_g2_i4 | Glutathione S-transferase | GSTM2_MOUSE Glutathione S-transferase Mu 2 OS=*Mus musculus* | PREDICTED: *Girardinichthys multiradiatus* glutathione S-transferase Mu 1-like (LOC124884942), mRNA |
| TRINITY_DN49_c0_g1_i1 | Lipocalin-like domain | CRA2_HOMGA Crustacyanin-A2 subunit OS=*Homarus gammarus* | PREDICTED: *Procambarus clarkii* crustacyanin-A2 subunit (LOC123770817), transcript variant X3, misc_RNA |
| TRINITY_DN19741_c0_g1_i1 | Adipokinetic hormone | RPCH_PENMO Red pigment-concentrating hormone OS=*Penaeus monodon* | *Cherax quadricarinatus* red pigment-concentrating hormone (RPCH) mRNA, complete cds |
| TRINITY_DN3588_c0_g1_i2 | Lipoprotein amino terminal region | VIT_PENME Vitellogenin OS=*Penaeus merguiensis* | *Homarus americanus* vitellogenin mRNA, complete cds |
| TRINITY_DN3862_c0_g1_i4 | Low-density lipoprotein receptor domain class | VLDLR_MOUSE Very low-density lipoprotein receptor OS=*Mus musculus* | PREDICTED: *Cherax quadricarinatus* low-density lipoprotein receptor 2-like (LOC128703600), transcript variant X5, mRNA |
| TRINITY_DN963_c1_g1_i5 | Flotillin | FLOT1_DROME Flotillin-1 OS=*Drosophila melanogaster* | - |
| TRINITY_DN4211_c0_g1_i9 | Retinal pigment epithelial membrane protein | BCDO2_MOUSE Beta,beta-carotene 9',10'-oxygenase OS=*Mus musculus* | - |
| TRINITY_DN1004_c0_g1_i2 | Retinal pigment epithelial membrane protein | BCDO1_HUMAN Beta,beta-carotene 15,15'-dioxygenase OS=*Homo sapiens* | - |
| TRINITY_DN2509_c0_g1_i1 | Retinal pigment epithelial membrane protein | NINAB_GALME Carotenoid isomerooxygenase OS=*Galleria mellonella* | - |
| TRINITY_DN39850_c0_g1_i1 | - | CYB_APOGR Cytochrome b OS=*Apomys gracilirostris* | *Adineta vaga* cytochrome b (CYTB) gene, complete cds; mitochondrial |
