# Supplementary material for: Enhancing the color and stress tolerance of cherry shrimp (Neocaridina davidi var. red) using astaxanthin and Bidens Pilosa
Source: PLoS One. 2024 Dec 19;19(12):e0315585. doi: 10.1371/journal.pone.0315585 (PMC11658619; doi:10.1371/journal.pone.0315585)
Supplement: S2 Table — (DOCX) [file pone.0315585.s005.docx]

**S2 Table. Annotation database for emergency -related genes in the Neocaridina shrimp transcriptome.**

| **Transcript_ID** | **Pfam** | **Swiss – Prot** | **NCBI BLAST** |
| --- | --- | --- | --- |
| TRINITY_DN1624_c0_g1_i1 | Catalase | CATA_MOUSE Catalase OS=*Mus musculus* | *Macrobrachium rosenbergii* catalase mRNA, complete cds |
| TRINITY_DN74904_c0_g1_i1 | Iron/manganese superoxide dismutases, alpha-hairpin domain | SODM1_CAEEL Superoxide dismutase [Mn] 1, mitochondrial OS=*Caenorhabditis elegans* | *Homo sapiens* superoxide dismutase 2 (SOD2), transcript variant 8, mRNA; nuclear gene for mitochondrial product |
| TRINITY_DN1527_c1_g1_i9 | HIF-1 alpha C terminal transactivation domain | HIF1A_HUMAN Hypoxia-inducible factor 1-alpha OS=*Homo sapiens* | *Palaemon carinicauda* hypoxia-inducible factor 1 alpha mRNA, complete cds |
| TRINITY_DN580_c0_g1_i3 | Histidine kinase-, DNA gyrase B-, and HSP90-like ATPase | HS90A_RABIT Heat shock protein HSP 90-alpha OS=*Oryctolagus cuniculus* | *Anaphothrips obscurus* heat shock protein 90 mRNA, complete cds |
| TRINITY_DN5642_c1_g2_i4 | Glutathione S-transferase | GSTM2_MOUSE Glutathione S-transferase Mu 2 OS=*Mus musculus* | PREDICTED: *Girardinichthys multiradiatus* glutathione S-transferase Mu 1-like (LOC124884942), mRNA |
